# How diversification of overseas subsidiaries affects parent company innovation performance: evidence from China’s multinationals

**DOI:** 10.3389/fpsyg.2024.1344816

**Published:** 2024-05-06

**Authors:** Siyi Wang, Xinni Chen, Jinsong Ye, Changbiao Zhong

**Affiliations:** Business School, Ningbo University, Ningbo, China

**Keywords:** diversification of overseas subsidiaries, innovation performance, overseas and domestic investment layout, Chinese multinationals, outward foreign direct investment (OFDI)

## Abstract

This paper examines the diversification of overseas subsidiaries on innovation performance of the parent company. Based on theoretical analysis and a combined Chinese firm dataset from 2000 to 2013, we find that diversification of overseas subsidiaries positively promotes the parent company innovation performance through the spillover effect of innovation capabilities. In addition, we determine that both the overseas and domestic investment layout can positively moderate the main effect. But there are differences between them. In concrete terms, the domestic investment layout plays a substitution effect in developed areas and acts a more pronounced moderating role in state-owned sample. Besides, the overseas investment layout plays a more important substitutive moderating role on non-state-owned enterprises. This research provides a special insight for studying the reverse spillover effect of OFDI in terms of the contribution of subsidiary linkages and offers several recommendations for multinational corporations to enhance the global competitiveness.

## 1 Introduction

Against the backdrop of new changes in globalization especially under the shock of geopolitical games and partial wars, multinational corporations will choose to diversify their investment layout and embed themselves deeply into global value chains to tackle operational and security risks. In the process of globalization, the strategic choices of multinational corporations are highly differentiated and are subjective decisions based on their own understanding in response to the changes of objective constraints. A homogenous investment layout may place the company at greater risk, while a diversified investment layout is equivalent to “putting eggs in different baskets,” which can enhance a company’s ability to survive and thrive.

Innovation performance represents the innovation outcomes achieved by companies and is critical to the resilience to risk and sustainability of global competitiveness. Multinational corporations are the major players in international direct investment. Since the beginning of this century, multinational corporations from Asia have established a large number of overseas subsidiaries and improved their own innovation performance in the meantime. The Uppsala Model (Johanson and Vahlne, 1977) provides a theoretical basis for enterprises that lack inherent advantages in internationalization to make outward foreign direct investment. Many industry cases and academic studies have confirmed that outward foreign direct investment is increasingly becoming an important channel for multinational corporations to enhance their innovation performance, thus cultivating risk-resilient core competencies (Akben Selçuk, 2015; Akcigit et al., 2016; Piperopoulos et al., 2018; Huang et al., 2020; Lee et al., 2023; Dai et al., 2024). Multinational corporations investing abroad, especially in countries or regions with high levels of innovation, can benefit from the host country’s advantages to complement their own development; overseas subsidiaries or branches can obtain and utilize resources to benefit the parent corporation, thereby generating a reverse spillover effect.

According to the 2022 Statistical Bulletin of China’s Outward Foreign Direct Investment, China has been ranked among the top three global OFDI flows for 11 consecutive years, and its contribution to the world economy has become increasingly prominent. Chinese enterprises are actively seeking new markets, resources and technologies overseas to promote the internationalization. In parallel, the modes and targets of OFDI are evolving, from simple resource acquisition or market expansion to a gradual integration of technology innovation, brand building and global management. Chinese multinational corporations have benefited from the rapid growth and internationalization of the Chinese economy. These companies have gradually spread from the Chinese market to the international market and have become important contributors to the global economy. Until now, more than one-third of the world’s top 500 multinationals are from emerging economies represented by China. For example, Huawei, as China’s most globalized company with subsidiaries all over the world, is supposed to be one of the most skilled Chinese multinationals at the diversification of their overseas layout. Different subsidiaries with different locations serve different functions. According to the 2022 annual report^1^, Huawei International Pte. Ltd. in Singapore is mainly responsible for the purchase and sale of communication products, Huawei Technologies Japan K.K. is mainly responsible for R&D, sale and ancillary services of communication products, and Huawei Technologies Coöperatief U.A. in the Netherlands often acts as an intermediary role for certain overseas subsidiaries. Therefore, relying on such diversification advantages of the overseas subsidiaries, Huawei can obtain more resources and technical support through cooperation with local enterprises and suppliers, as well as the rich research resources and innovation environment, so as to better promote product innovation and technology advancement, and enhance its competitiveness in the international market. When a multinational corporation arranges its overseas subsidiaries, it will choose a specific destination or multiple destinations, some of which may be invested in more than once. What is more, these subsidiaries often take on one or more functions, which may be production, R&D, or a combination of both and even other functions. These overseas subsidiaries, which are positioned for different strategic functions, may have cooperative or even competitive relationships with each other. According to the theory of reverse spillovers (Chen et al., 2012; He, 2023), companies from less developed economies investing in companies from developed economies are able to gain novel resources and technology enhancement, which in turn contributes to the development of their parent firms in the home country, so interactions among the individuals brought about by the diversification of overseas subsidiaries can have an impact on the innovation performance of the parent company.

There are three main aspects of the importance of studying the effects of subsidiary diversification on innovation performance, which can also fill the gap in the existing literature and provide new insights and understanding to both academia and industry. First, understand the effects of firms’ diversification strategies better. Firms use subsidiary diversification strategies to expand their business scope and reduce risks, but the actual effects of such strategies on innovation performance may not be fully understood. Therefore, studying this topic helps us to better understand the effects of corporate diversification strategies and provides a theoretical basis for corporate strategic decision-making (Dai et al., 2024; Wang et al., 2024). Second, promote the development of innovation theory. Innovation is a critical driver of sustained competitiveness of enterprises. By studying the impact of subsidiary diversification on innovation performance, we can gain a deeper understanding of the mechanism of internal and external factors in the innovation process, promote the development of innovation theory, and provide more effective innovation strategies for enterprises (Ferraris et al., 2020; Audretsch et al., 2024). Third, offer a perspective to the existing controversy of diversification strategy. There is a dispute about the benefits and costs of company diversification strategy. Some studies have pointed out that diversification strategies may disperse firms’ resources and reduce innovation performance (Papanastassiou et al., 2020). Therefore, examining the impact of subsidiary diversification on innovation performance can help resolve the controversy over diversification strategies and provide more accurate guidance for company strategy formulation.

In view of the importance of overseas subsidiary diversification on company innovation performance and the representativeness of the China’s OFDI sample, this study mainly explores the impact of diversification of overseas subsidiaries on the innovation performance of the parent company using Chinese firm level data from 2000 to 2013. Furthermore, this paper also explores the moderating effects of overseas investment layout and domestic investment layout. Considering the focus on investigating the overseas learning opportunities of subsidiaries, examining the moderating effects of overseas investment layout becomes particularly crucial. Emerging market enterprises often encounter various challenges and opportunities in the international market, especially in different countries and regions’ competitive environments, where the overseas investment layout may have complex and diverse effects on a company’s innovation performance. Moreover, it is noteworthy that Chinese enterprises mostly lack the monopolistic advantages outlined in traditional international investment theories. Therefore, this paper innovatively proposes that experiences gained from domestic cross-regional investments within a large economy can assist companies in overcoming the liability of foreignness encountered as newcomers. Enough learning experiences from domestic investment can facilitate resource allocation, technology transfer, and innovation capabilities within the parent company, thus playing an important role in driving the reverse technology spillover effect of overseas subsidiaries.

This paper has two main theoretical contributions. First, exploring the impact of diversification on innovation performance from the perspective of subsidiaries. Most of the previous studies have focused on the whole parent company level, but this research explores the impact of inter-individual linkages on the innovation performance of parent firms from the perspective of overseas subsidiaries, deepens the research on reverse spillovers, and enriches the theory of outward foreign direct investment (OFDI) in emerging economies represented by China, as distinguished from the traditional theory of OFDI under the framework of neoclassical economics that focuses on multinational corporations in developed countries. Second, the overseas investment layout and domestic investment layout are jointly included in the analytical framework. Most of the previous literature on OFDI always focuses on external factors but ignores the effect of domestic experience (Ferraris et al., 2020; Audretsch et al., 2024), and we try to fill the gaps in this part of the literature. By placing these two types of investment layouts in the same analytical framework, it is possible to better compare their impact on innovation performance, reveal their interactions and possible substitutions, and contribute to a more comprehensive understanding of the relationship between the strategic choices of multinational corporations and their innovation performance.

The remainder of the paper is organized as follows. Section 2 presents an overview of relevant literature strands. Section 3 discusses the theoretical analysis on how diversification of overseas subsidiaries influences parent company innovation performance and proposes the empirical hypothesis. Section 4 describes the research methods, including data, model and variables. Section 5 reports and analyzes the empirical results. Section 6 concludes the paper, including the limitations and prospects.

## 2 Literature review

Our paper is based on three branches of literature: one is related to the reverse spillover effect of OFDI, another is related to studies on overseas subsidiaries of multinational corporations, and the last is related to the impact of diversification of overseas subsidiaries on company innovation performance. We have briefly summarized the relevant content of the main literature in Table 1.

**Table 1:** The summary of relevant main literature.

Branches	Source	Method / Data	Findings
The reverse spillover effect of OFDI	Bitzer and Kerekes (2008)	Manufacturing-level data from 17 OECD countries	No evidence of positive impact of outward investment on international knowledge transfer.
	Masso and Vahter (2012)	Firm-level data in Estonia	Foreign direct investment activities have a significant contribution at the firm level, but no visible impact at the country level.
	Piperopoulos et al. (2018)	High-tech companies in China	Foreign investment destined for developed economies contributes more significantly to the level of technological innovation of domestic firms.
	Fu et al. (2022)	Firm-level data	The presence of reverse spillovers is confirmed.
	Kong et al. (2024)	Firm-level data	OFDI increases the level of domestic human capital and the intensity of R&D, narrowing the technology gap.
Studies on overseas subsidiaries of multinational corporations	Kafouros et al. (2012)	Firm-level data	Geographic breadth and depth of foreign subsidiaries are correlated with firms’ innovation performance.
	Deng et al. (2020)	Theoretical research	The increase in geographic breadth is more positively correlated with parent firm innovation performance.
	Luo et al. (2021)	Firm-level data	Geographic diversification of overseas subsidiaries has significantly increased the company’s productivity.
	Mao et al. (2023)	High-tech companies in China	Geographically decentralized foreign subsidiaries can facilitate knowledge spillovers.
The impact of diversification of overseas subsidiaries on innovation performance	Feldman (2014)	Theoretical research	In the process of diversification of enterprises, information asymmetry is a major obstacle to their performance improvement.
	Lee (2023)	Firm-level panel data	Firms prefer the convergent strategy to maximize their value.
	Dai et al. (2024)	Firm-level data	Increased technology-related investment across regions will contribute to broadening the horizon of knowledge, increasing the risk resilience of firms, and leading to innovation.

### 2.1 The reverse spillover effect of OFDI

At an early stage, some scholars were skeptical about the existence of spillover effects, arguing that the overseas investments of multinational corporations might not have a positive impact on their home countries (Bitzer and Kerekes, 2008; Masso and Vahter, 2012). However, with deeper research and accumulation of data, more and more studies have found positive spillover effects of multinational corporations’ overseas investment (Piperopoulos et al., 2018; Fu et al., 2022). Many scholars have noted the learning and interaction process of the multinationals in their overseas investment activities, which can be broadly categorized into four aspects. Firstly, overseas investment by multinationals often brings advanced technology and management experience (Hu et al., 2021; Huang and Huang, 2023; Kong et al., 2024), and such technology diffusion not only improves the productivity and product quality of the parent company in the home country, but also stimulates innovative activities, thus promoting the technology level and innovation ability of enterprises. Secondly, multinationals often cooperate and communicate with local enterprises in their overseas investment activities, sharing management experience, marketing strategies, technical talents and other resources (Audretsch et al., 2024; Wang et al., 2024). This kind of knowledge sharing not only helps to improve the management level and market development of the parent company, but also promotes the cultivation of the talents and the inheritance of technical skills (Kong et al., 2024). Also, when multinationals engage in overseas investment activities, they often expand new markets (Nire and Matsubayashi, 2024) and supply chains by setting up overseas production bases and sales networks, so as to improve the market coverage and competitiveness of their products. Moreover, by establishing production bases and R&D centers in different countries, multinationals can achieve optimal allocation of resources and synergistic development of the industry chain (Xie et al., 2019; Chen et al., 2023; Kong et al., 2024), thus enhancing the efficiency and competitiveness of the parent company and even the global economy.

Based on the review of the above literature, this paper believes that foreign direct investment corporations in emerging economies conduct capital export activities in developed economies, which is equivalent to opening up a channel to obtain technology, markets and specific resources in the host country, absorbing excellent elements through this channel, and then transform them into the parent company’s own competitiveness.

### 2.2 Studies on overseas subsidiaries of multinational corporations

How multinational corporations lay out their overseas subsidiaries has attracted broad academic attention. In previous studies, scholars usually regarded the overseas layout of multinational corporations as simple geographical expansion, focusing on factors such as the geographical location (Kafouros et al., 2012; Deng et al., 2020; Luo et al., 2021; Mao et al., 2023), market share (Lenihan et al., 2023) and access to resources of subsidiaries (Wang and Zhang, 2023; Gurkov and Morley, 2024). However, with the acceleration of globalization and the increasing competition in the market, more and more scholars have begun to realize that the functional positioning of subsidiaries is crucial to the long-term competitive advantage of enterprises (Meyer et al., 2020; Papanastassiou et al., 2020; Christofi et al., 2021; Brock and Hitt, 2024). Functional positioning covers not only the roles and functions of subsidiaries in production, sales and services, but also their position and role in the global value chain. By studying the functional positioning of subsidiaries, the strategic objectives and international competitive strategies of the multinationals can be better understood. For example, some subsidiaries may be positioned as production bases, responsible for producing and manufacturing products, while others may be tasked with sales and market expansion. Such different functional positioning reflects the parent company’s strategic choices and layout for different markets and business environments.

Unfortunately, however, most of the studies on overseas subsidiaries mainly focus on the geographical location aspect, while the functional positioning aspect does not seem to have attracted much attention from scholars. This is important, however, because the functional positioning of subsidiaries directly reflects the strategic needs of the parent company and allows for a better understanding of corporate internationalization. Therefore, this paper attempts to contribute to this branch of the literature.

### 2.3 The impact of diversification of overseas subsidiaries on innovation performance

The above literatures further examine the reverse spillover effect of outward FDI setting diversified overseas subsidiaries and affiliates, but most of them consider the impact of a single view from the perspective of the parent company, and few literatures take diversified subsidiaries especially functional diversification into consideration. Inspired by the ideas of Busenbark et al. (2022) and Lee (2022), this paper attempts to study the impact of diversification of overseas subsidiaries on parent company innovation performance.

Many articles analyze the impact of diversification on the innovation performance from the corporate management and business operation aspects (Feldman, 2014; Lee, 2023), but most of them consider the impact from the perspective of the whole parent company. Therefore, this paper attempts to study the impact of diversification of overseas subsidiaries on parent company innovation performance from the perspective of subsidiaries. Diversification of subsidiaries not only brings more market opportunities and resources to the parent company, but also promotes the parent company’s innovation performance through innovative activities in different fields (Bodlaj et al., 2020; Dai et al., 2024; Sierra-Morán et al., 2024). For example, the establishment of diversified subsidiaries overseas by some multinational corporations can provide better access to local market information and technological resources (Dai et al., 2024), thus accelerating the development and promotion of new products. In addition, diversification of subsidiaries can also promote knowledge exchange and technological innovation within the firm and enhance the innovation capability and competitiveness of the whole firm (Kong et al., 2024). However, diversification of subsidiaries may also bring some challenges and risks (Ferraris et al., 2020; Papanastassiou et al., 2020). For example, there may be resource competition and conflict of interest among subsidiaries in different fields, resulting in the parent company’s innovation activities being restrained. In addition, diversification of subsidiaries may also increase the management costs and risks of the firm and reduce overall innovation performance.

## 3 Theoretical analysis and hypothesis

### 3.1 Impact of diversification of overseas subsidiaries

Traditional research usually believes that enterprises are independent of the market to make decisions, and regards those subsidiaries as a whole. But in fact, the enterprise’s decision-making is not the result of its independent thinking, but also affected by the group in a similar environment, there will be interaction and learning between each subsidiary (Ellison, 1993). When multinationals make outward foreign direct investment, they may make one or more investments in one or more host countries based on different investment objectives, and these overseas subsidiaries, due to their different investment motives and strategic positioning, assume one or more functions, and there are some relationships among them. Overseas subsidiaries of multinationals carry out the activities of acquiring, digesting, assimilating, integrating and transferring innovation resources, and at the same time accessing various types of opportunities through external networks with consumers, suppliers and competitors in the host country, thus promoting innovation performance. Unlike previous studies that emphasized the geographic distribution of subsidiaries, diversification in terms of functional positioning can help form stable and mature overseas investment networks, and the network model can ensure a good corporate governance commitment mechanism (Siegel, 2009). Diversification can enable the company to not only seek knowledge and other resources, but also make full use of the host country resources as well as learn from various types of knowledge to influence the continuity of the network and improve the quality of information, which is crucial for innovation performance. Specifically, on the one hand, functionally diversified overseas subsidiaries can operate in different fields or markets, which can facilitate cross-field flows of technology and knowledge (Papanastassiou et al., 2020; Huang and Huang, 2023; Kong et al., 2024); on the other hand, these overseas subsidiaries are able to cooperate with different partners and suppliers, often sharing resources, experience and technology, which can lead to more abundant external resources and cooperation opportunities (Ferraris et al., 2020). As a result, when subsidiaries have accumulated certain specialized knowledge and experience, these resources can be diffused to other subsidiaries or the parent company through the company’s internal cross-sector network, thus facilitating indirect learning and knowledge absorbing within the firm. This cross-field flows of knowledge, experience and resources help to break down the barriers and promote innovative activities, which in turn enhance the innovation performance. For example, a subsidiary with R&D function aims to make full use of innovation resources in the host country. While the parent company may be able to profit from knowledge and other resources through reverse technological spillovers, it may not be able to truly understand where innovations come from and how they can be applied to the production process, and it is only by fully embedding itself in the local production and R&D networks that it can truly understand and identify useful resources and make effective use of them.

Therefore, when companies engage in diversifying their overseas subsidiaries, they will expand their operations into different regions or markets and collaborate with local partners. This diversification strategy not only brings new markets, resources, and opportunities to the companies but also facilitates the exchange of technology and accumulation of cross-disciplinary knowledge. Through interactions with local partners, companies will gain access to specialized knowledge and experience from various fields, thereby enhancing their innovation capabilities (Nascimento et al., 2024). The accumulation of cross-disciplinary knowledge and experience enables companies to break through industry and technological barriers and transcend traditional modes of thinking by integrating best practices and technologies from different domains into their innovation activities (Pattinson and Dawson, 2024). Such cross-disciplinary innovation capabilities often lead to more forward-looking (Verreynne et al., 2023) and disruptive solutions, thereby granting companies a competitive advantage in the market. Moreover, the spillover of innovation capabilities is crucial for companies as it not only facilitates the dissemination, sharing, and application of knowledge and technology but also contributes to the construction of an internal innovation ecosystem within the organization (Battistella et al., 2023; Wang et al., 2023). This innovation ecosystem, fostered through the integration of internal and external resources, drives the sustained development of innovation activities. In summary, diversification of overseas subsidiaries provides companies with abundant resources and opportunities, and promotes the accumulation of cross-disciplinary knowledge and experience, strengthening the spillover effects of innovation capabilities. These spillover effects not only drive continuous innovation activities but also enhance companies’ innovation performance, enabling them to achieve enduring competitive advantages in the market.

Based on this, the following assumptions are proposed:

*Hypothesis 1a*. The diversification of overseas subsidiaries has a positive effect on parent company innovation performance.

*Hypothesis 1b*. The diversification of overseas subsidiaries enhances the innovation performance of the parent company through the spillover effects of innovation capabilities.

### 3.2 Moderating role of overseas and domestic investment layout

While diversification of overseas subsidiaries can facilitate the acquisition of cross-sector knowledge, enhance internal organizational coordination as well as promote resource allocation and sharing, excessive overseas investment layout may reduce the synergy effect among subsidiaries. If a multinational company invests extensively in multiple countries, the subsidiaries in each country may face different markets, cultures, and legal environments, leading to diminished synergies within the company (Batsakis et al., 2018; Meyer et al., 2020). For example, subsidiaries may have competitive rather than cooperative relationships with each other, which may hinder the sharing of knowledge and experience so that the parent company is unable to effectively learn and absorb knowledge from its subsidiaries, thus hampering the enhancement of innovation performance. Some studies have shown that the overseas investment layout impacts the capital allocation ability of enterprises from the perspective of breadth and depth (Kafouros et al., 2012; Love et al., 2014), and others have found that they are closely related to the innovation of multinational enterprises (Martin and Salomon, 2003; Lai et al., 2010). Particularly, the breadth refers to the number of countries or regions of the investment destinations and the depth refers to the number of times the investment is made within a single market. From the perspective of the breadth of foreign direct investment, enterprises can access the factor endowment advantages of different markets, and diversified incentives can be better embedded in different markets by making full use of the resources of each market. However, with the continuous improvement of breadth, the corporation faces a higher liability of foreignness, owing to operations in different markets, and the cost of coordination and communication between subsidiaries and the parent corporation increases (Castellani et al., 2017). It is also unable to better identify and use various types of knowledge in the host country, inhibiting the diversification of overseas subsidiaries. Judging from the depth of foreign direct investment, although centralized operations in a few markets are beneficial for enterprises to digest and absorb relevant resources, the limited resources may restrain the effect of diversification of overseas subsidiaries.

In contrast, if multinationals concentrate more of their investments in the domestic market, more synergies between subsidiaries can be created, which can enhance the positive effects. When a multinational enterprise operates across regions within its home country, it can deal with problems that may arise when the market is unfamiliar, and is better equipped at managing consumers, suppliers, and competitors in different domestic markets. The accumulated experience of operating in multiple markets and being concentrated in a few markets in the home country can help enterprises to overcome the liability of foreignness. On the one hand, subsidiaries may gain easier market access in the domestic market, as they may have a better understanding of the local market environment and regulations and be better able to adapt to the local culture and consumer needs. The easier market access provides more opportunities for subsidiaries to acquire local market knowledge and information, including consumer feedback, competitor dynamics, and supply chain operations (Dai et al., 2024). This local market knowledge and information can be transferred to the parent company through the subsidiary, thereby enriching the parent company’s knowledge base and contributing to its innovation performance. On the other hand, subsidiaries may have easier access to local resource support, such as talent, supply chain, and partners (Kong et al., 2024). These supports can help subsidiaries conduct their investment more effectively and provide more collaboration opportunities to the parent company. For example, a subsidiary may partner with local universities to recruit talented people for R&D, or establish partnerships with local suppliers to ensure a stable and efficient supply chain (Meyer et al., 2020). These resource supports not only improve the competitiveness of the subsidiary, but also transfer through the subsidiary to the parent company, thus contributing to the parent company’s ability to innovate.

Therefore, the following assumptions are proposed:

*Hypothesis 2a*. The overseas investment layout has a negative moderating effect.

*Hypothesis 2b*. The domestic investment layout has a positive moderating effect.

## 4 Methods

### 4.1 Data and sample

Aiming at the impact of the diversification of overseas subsidiaries on the parent company innovation performance, this paper has adopted a panel dataset. Due to the quality and availability of data, we choose the Chinese industrial enterprises database, the list of overseas investment enterprises (institutions) of the Ministry of Commerce of China (the following is referred to as the outward investment list), and the Chinese patent database. Chinese industrial enterprise database is sourced from the National Bureau of Statistics and is the raw data at the enterprise level, with statistics targeting large and medium-sized manufacturing enterprises with an annual turnover of 5 million yuan and above. It is one of the most comprehensive enterprise databases, offering advantages such as large sample size, long duration, and diverse indicators, providing a good reflection of enterprise conditions. The main variables cover enterprise basic indicators, like enterprise name, industry, opening year, employee number, and enterprise financial indicators, like fixed assets, main business income, R&D expenses, and total industrial output value. Following the approach of existing literature (Brandt et al., 2012; Huang et al., 2020), we excluded samples with missing or negative values for four variables: total output, intermediate input, capital stock, and industrial value added. Additionally, samples with missing or fewer than 8 employees were also excluded. Besides, the outward investment list mainly selects the year and business scope of each foreign investment of the corporation, and the patent database primarily selects information about the number of applications, authorizations, cited numbers of invention patents, utility models, and design patents of the corporation. In addition, the data on domestic investment layout are mainly obtained from the “Qcc.com” platform, which manually matches foreign investment enterprises with their domestic subsidiaries. We have matched the above databases based on the names of domestic parent companies and the years, resulting in a panel dataset spanning from 2000 to 2013, with a total of 7,299 parent companies participating in outward investment, which provides sufficient support for our study and still holds value today. Because during that period, Chinese enterprises experienced a phase of internationalization following China’s accession to the WTO, fostering extensive engagements with globalized enterprises and cultivating a strong inclination for global expansion. Enterprises had various purposes for going global, including the desire to acquire advanced knowledge, which is even more crucial for Chinese enterprises in the contemporary era. Currently, globalization is undergoing new changes, as numerous enterprises from emerging economies are venturing into new global layout patterns. Therefore, analyzing the samples of Chinese enterprises’ global expansion from 2000 to 2013 still has high reference value for enterprises from other new economy entities in today’s world.

### 4.2 Model specification

Based on the previous theoretical analysis, we have constructed the following empirical models:


(1) $$\begin{array}{l} \text{Innovation }{\text{performance}}_{it}=\\ \alpha_{0}+\alpha_{1}\text{Divers}e_{it}+\alpha_{2}\text{Oversea}\_{\text{lay}}_{it}\\ +\alpha_{3}\text{Domes}\_{\text{lay}}_{it}+X_{it}+\left\{F\right\}+\varepsilon_{it} \end{array}$$

(2) $$\begin{array}{l} \text{Innovation }{\text{performance}}_{it}=\\ \alpha_{0}+\alpha_{1}\text{Divers}e_{it}+\alpha_{2}\text{Oversea}\_{\text{lay}}_{it}+\\ \alpha_{3}\text{Domes}\_{\text{lay}}_{it}+\alpha_{4}\text{Diverse}\_\text{overse}a_{it}+\\ \alpha_{5}\text{Diverse}\_\text{dome}s_{it}+X_{it}+\left\{F\right\}+\varepsilon_{it} \end{array}$$

In the above model, i and t represent the parent company and observed year respectively, and α is the estimation parameter. $\text{Innovation }{\text{performance}}_{it}$ refers to a parent corporation’s innovation performance. $\text{Divers}e_{it}$ refers to the diversification of overseas subsidiaries. $\text{Oversea}\_{\text{lay}}_{it}$ is the moderating variable, representing the layout of overseas investment. $\text{Domes}\_{\text{lay}}_{it}$ indicates the layout in the domestic investment market. And $\text{Diverse}\_\text{overse}a_{it}$ is the interaction term of the diversification of overseas subsidiaries and the overseas investment layout, while $\text{Diverse}\_\text{dome}s_{it}$ is the interaction term of the diversification and the domestic investment layout. Besides, X represents the control variables at the enterprise and national levels. {F} represents the model’s impact on province, industry, year, and firm-level fixed effects. $\varepsilon_{it}$ is a random perturbation term. Although we have added the province fixed effect in the current model to mitigate biases stemming from factors such as regional economic development level and policy environment, introduced the industry fixed effect to control differences among industries in technology level, market demands, and competitive intensity, included the year fixed effect to capture the temporal trends affecting the results, and incorporated the firm fixed effect to diminish potential biases arising from firm heterogeneity, we cannot entirely eliminate other possible sources of biases. For instance, the model may be affected by reverse causality, where the innovation performance of the parent company may influence the diversification of overseas subsidiaries. Therefore, in order to enhance the reliability and persuasiveness of the results, we will conduct further robustness tests and endogenous treatments in the subsequent sections.

Formula (1) denotes the effect of diversification of overseas subsidiaries, the core explanatory variable, on the innovation performance of the parent company. If $\alpha_{1}$ is significantly positive, it indicates that the diversification of overseas subsidiaries has a positive effect on the parent corporation’s innovation performance. Formula (2) explores the moderating effects of overseas and domestic investment layouts. If the estimation parameter is significantly positive, the corresponding moderating variable has a positive moderating effect; while if the estimated parameter is significantly negative, the corresponding regulatory variable has an inhibitory effect.

### 4.3 Measurement of variables

#### 4.3.1 Innovation performance

The dependent variable *Innovation Performance* is defined as the parent company innovation performance, which is represented by the logarithm of the sum of the flowing data of patent grant, including invention patents, utility models, and design patents (Hu et al., 2017; Jiang et al., 2020).

#### 4.3.2 Diversification of overseas subsidiaries

The explanatory variable *Diverse* is defined as the diversification of overseas subsidiaries, which is usually measured either as a continuous variable (Maggio and Sitko, 2021; Nigam and Gupta, 2021) or as a binary variable (Bartolini et al., 2014; Jouida and Hellara, 2018). First, we take a continuous variable as a measurement in the baseline regression. Referring to Lu and Beamish (2004), we count the number of overseas subsidiaries and the number of host countries or regions involved in the investment of each company in each year, and then following the procedure of Sanders and Carpenter (1998) to, respectively, divide the number of both by the maximum value of each in the whole sample to obtain the two proportions, and then calculate their mean values. The final result is the degree of diversification of overseas subsidiaries, which is between 0 and 1. Second, we have arranged a binary variable to replace the original measurement in the robustness test. We use keywords to search the business scope of corporations in the investment list in order to classify the functions of the subsidiaries. For example, those with keywords such as production, manufacturing, processing and assembly are classified as production function; those with keywords such as research and development, research and development, technology development and material development are classified as R&D function; those with keywords such as sales, distribution, development, promotion and business expansion are classified as market function; the rest are classified as other functions. Those with only one function are classified as no diversification and the value is zero; those with two or more functions are classified as diversification and the value is one.

#### 4.3.3 Control variables

The control variables include both firm level and national level. The variable *Age* is defined as the firm age, which is calculated by subtracting the year of opening from the year of observation and the year of opening takes the value of zero. Following the existing literature (He and Wong, 2004; Wu et al., 2020), we have took the logarithm transformation to reduce the potential volatility. *Size* is measured by the logarithm of the number of employees in the corporation (Dzeraviaha, 2023). *Productivity* is measured by the logarithm of the ratio of gross industrial output value to the number of employees (Yao, 2015). *RD_intensity* is measured by the ratio of new product output to industrial output to measure (Liu et al., 2023). *Manage* is defined as the managerial capability of the firm, which is represented by the ratio of main business income to total assets (Wu and Yang, 2023). *Experience* is defined as the overseas experience of the company, which is measured by year of observation minus year of initial outward FDI (Batsakis et al., 2018). *State_owned* represents the proportion of state capital in the total assets of the selected sample (Suu et al., 2021). *Internationalization* refers to the average of the ratio of foreign assets to total assets and the ratio of foreign sales to total sales (Kafouros et al., 2012). *Distance* is measured by taking the logarithm of the geographic distance between the most populous city in the country where the parent company and the corresponding overseas subsidiary are located (Goetz and Morschett, 2023).

#### 4.3.4 Moderating variables

The moderating variables include overseas investment depth and domestic investment breadth. *Oversea_lay* is defined as overseas investment depth, which is measured by the number of times a firm has invested in the overseas market (Zhang et al., 2024). *Domes_lay* is defined as domestic investment breadth, which is measured by the parent company’s total investment in all domestic subsidiaries divided by the amount of subsidiaries’ locations (Chen et al., 2023).

Table 2 presents the descriptive statistics of the variables. The standard deviation values are all located between 0 and 5, indicating that the sample distribution tends to be concentrated and the data stability is relatively good.

**Table 2:** Descriptive statistics of variables.

Variable type	Variable name	Obs.	Mean	S.D.	Min	Max
Dependent variable	Innovation Performance	55,312	0.731	1.288	0	9.041
Explanatory variable	Diverse	55,312	0.071	0.111	0.031	1
Control variable	Age	55,312	2.244	0.708	0	4.159
	Size	54,942	6.074	1.485	2.197	12.580
	Productivity	54,942	6.130	1.213	0	16.400
	RD_intensity	27,648	0.123	0.290	0	5.251
	Manage	53,843	1.711	2.694	0	150.100
	State_owned	41,041	0.056	0.197	0	1
	Experience	46,345	3.228	2.700	0	25
	Internationalization	40,376	0.253	0.286	0	2.595
	Distance	46,345	8.466	0.776	6.698	9.868
Moderating variable	Oversea_lay	46,345	0.515	0.581	0	7
	Domes_lay	9,614	3.646	4.430	1	49

## 5 Empirical results analysis

### 5.1 Results of baseline regression

Based on the models and variables described in the previous text, we have conducted the baseline regression. Table 3 reports the baseline regression results. Column 1 simply regresses the explained variables and explanatory variables, column 2 adds control variables based on column 1, column 3 controls the year fixed effect, column 4 controls the year, province and firm fixed effects, and column 5 controls the year, industry and firm fixed effects. Column 6 controls the four fixed effects. The results of columns 1 to 6 show that the regression coefficients of the explanatory variables are significantly positive, which indicates that the diversification of overseas subsidiaries does have a positive effect on the innovation performance of the parent company, which is consistent with hypothesis 1a. Although the coefficients are all significant at the 1% level, there are differences in the coefficient values of column 1 to column 3 and column 4 to column 6. The reason is that the first three columns do not contain both time and individual fixed effects, which play a crucial role. Time fixed effects can control for time-dependent variation in the regression, helping us to capture the overall trend and impact of diversification of overseas subsidiaries on the innovation performance of the parent firm which changes over time, hence reducing the bias due to time variation. Besides, firm fixed effect can help us control the individual characteristics of firms that have not yet been observed and reduce inter-individual variation, allowing for a more accurate assessment of the main effect. Therefore, the coefficients on the main explanatory variables become stable when time and firm fixed effects are introduced in the last three columns. Taking column 6 as an example, the coefficient of the explanatory variable is 31.507, which means that the innovation performance of the parent company will increase by 31.507% for each unit increase in the diversification of overseas subsidiaries.

**Table 3:** Results of baseline regression.

	(1)	(2)	(3)	(4)	(5)	(6)
Diverse	3.962^***^	2.434^***^	2.691^***^	32.071^***^	31.507^***^	31.507^***^
	(0.046)	(0.169)	(0.169)	(4.601)	(4.528)	(4.528)
Age		0.196^***^	0.134^***^	0.333	0.443	0.443
		(0.036)	(0.036)	(0.461)	(0.560)	(0.560)
Size		0.327^***^	0.327^***^	0.022	0.082	0.082
		(0.018)	(0.017)	(0.133)	(0.139)	(0.139)
Productivity		0.473^***^	0.397^***^	0.156	0.217	0.217
		(0.026)	(0.027)	(0.128)	(0.154)	(0.154)
RD_intensity		0.440^***^	0.446^***^	−0.091	−0.049	−0.049
		(0.062)	(0.061)	(0.116)	(0.116)	(0.116)
Manage		−0.022	−0.058^**^	0.063	0.037	0.037
		(0.023)	(0.023)	(0.139)	(0.144)	(0.144)
State_owned		−0.497^***^	−0.251^***^	0.439	0.335	0.335
		(0.090)	(0.092)	(0.308)	(0.351)	(0.351)
Experience		0.037^***^	0.087^***^	0.060	0.050	0.050
		(0.009)	(0.011)	(0.038)	(0.042)	(0.042)
Internationalization		0.206^*^	0.257^**^	0.273	0.335	0.335
		(0.119)	(0.118)	(0.383)	(0.397)	(0.397)
Distance		0.024	0.021	0.002	0.002	0.002
		(0.032)	(0.032)	(0.002)	(0.002)	(0.002)
Oversea_lay		0.412^***^	0.284^***^	0.110	0.102	0.102
		(0.051)	(0.053)	(0.071)	(0.072)	(0.072)
Domes_lay		0.026^***^	0.034^***^	0.247^**^	1.230^**^	0.704
		(0.005)	(0.005)	(0.108)	(0.580)	(0.994)
Year	NO	NO	YES	YES	YES	YES
Province	NO	NO	NO	YES	NO	YES
Industry	NO	NO	NO	NO	YES	YES
Firm	NO	NO	NO	YES	YES	YES
Constant	0.448^***^	−5.588^***^	−6.800^***^	−7.368^***^	−9.967^***^	−2.736
	(0.006)	(0.347)	(0.405)	(1.819)	(2.612)	(6.305)
R-squared	0.117	0.358	0.383	0.748	0.757	0.757
*N*	55,312	2,855	2,855	2,855	2,855	2,855

*, ** and *** denote 10, 5 and 1% significance levels respectively, with standard errors in parentheses.

### 5.2 Robustness test

In this part, we have taken three approaches to conduct the robustness tests: replacing the dependent variable in two alternatives, replacing the explanatory variable, excluding the specific sample and replacing the database.

First, we replaced the dependent variable, using both the logarithm of the sum of the flowing data of patent citation and the inverse hyperbolic sine transformation of the value of the baseline regression (Ma et al., 2023) as alternative proxies for firm innovation performance. The corresponding results are shown in column 1 and column 2 of Table 4 respectively. The results of these tests are consistent with our original findings, indicating that our main results are robust to alternative measures of innovation performance, that is, the diversification of overseas subsidiaries does act a significant role on the parent corporation’s innovation performance.

**Table 4:** Results of the robustness test.

	(1)	(2)	(3)	(4)	(5)
Diverse	39.354^***^	37.904^***^	0.527^*^	30.605^***^	0.738^***^
	(3.807)	(5.052)	(0.279)	(4.843)	(0.145)
Age	−0.653	0.557	0.420	0.718	0.119^***^
	(0.424)	(0.652)	(0.546)	(0.684)	(0.007)
Size	0.122	0.075	0.089	0.051	0.001
	(0.123)	(0.160)	(0.137)	(0.170)	(0.003)
Productivity	0.071	0.260	0.214	0.192	0.010^***^
	(0.124)	(0.179)	(0.152)	(0.183)	(0.002)
RD_intensity	0.087	−0.066	−0.049	−0.115	0.019^***^
	(0.118)	(0.130)	(0.116)	(0.129)	(0.005)
Manage	0.032	0.018	0.038	0.045	−0.037^***^
	(0.105)	(0.146)	(0.144)	(0.157)	(0.013)
State_owned	−0.051	0.384	0.327	0.350	0.056
	(0.212)	(0.398)	(0.354)	(0.449)	(0.058)
Experience	0.052	0.050	0.047	0.049	0.005
	(0.032)	(0.045)	(0.042)	(0.052)	(0.007)
Internationalization	0.575^*^	0.308	0.339	0.598	−2.330^***^
	(0.346)	(0.430)	(0.405)	(0.600)	(0.054)
Distance	0.001	0.002	0.002	−0.004	−0.005
	(0.001)	(0.003)	(0.004)	(0.004)	(0.003)
Oversea_lay	0.027	0.117	0.111	0.084	0.001
	(0.062)	(0.081)	(0.070)	(0.077)	(0.004)
Domes_lay	2.006^***^	0.838	0.284	0.011	−0.151^***^
	(0.766)	(1.131)	(1.056)	(0.381)	(0.007)
Year	YES	YES	YES	YES	YES
Province	YES	YES	YES	YES	YES
Industry	YES	YES	YES	YES	YES
Firm	YES	YES	YES	YES	YES
Constant	6.273	−3.415	−0.527	−7.840^**^	1.416^***^
	(5.164)	(7.071)	(6.957)	(3.169)	(0.057)
R-squared	0.861	0.754	0.758	0.763	0.700
*N*	2,855	2,855	2,855	2,146	8,536

*, ** and *** denote 10, 5 and 1% significance levels respectively, with standard errors in parentheses.

Second, we replaced the explanatory variable with the binary values, which is represented in column 3 of Table 4. Despite replacing the measure of the diversification of overseas subsidiaries, its coefficient value is still significant. The results of these tests also support our original findings, further confirming the reliability of our empirical results.

Third, specific samples were removed. Previous studies (Tang et al., 2020; Han et al., 2024) generally regard the investment destined for Hong Kong as a springboard. Based on this, we excluded the Hong Kong sample from our analysis to assess the impact of this specific sub-sample on our results, which was shown in column 4 of Table 4. The exclusion of Hong Kong sample has not significantly altered our findings, indicating that our results are not driven solely by this particular sub-sample.

What’s more, we have made further examination using the matched data from the listed companies and the outward investment list. It is worth noting that our sample period has been extended to 2022 compared to the baseline regression, taking into account any changes that may have occurred due to the global pandemic in recent years. The results in the column 5 of Table 4 have shown that the coefficient for the diversification of overseas subsidiaries remained significantly positive at the 1% level, indicating the robustness of our findings and their ability to capture more recent trends.

### 5.3 Endogenous treatment

In addition to the robustness tests, we also conducted the endogenous treatment to address potential reverse causality issues. On the one hand, firm size is often recognized as an important factor influencing firms’ innovation performance, with significant differences between large and small and medium-sized firms in terms of resource allocation and market influence. Certain parent companies possess good innovation performance, which may not benefit from the diversification of overseas subsidiaries but from their own strength, in other words, there may be a possibility of reverse causality between diversification of overseas subsidiaries and the innovation performance of parent firms. On the other hand, the level of national development reflects the overall degree of development of a country in terms of economic, social and technological aspects, and there are obvious differences between countries at different development levels in terms of market size and technology level, which may interfere with the assessment of the role actually played by diversification of overseas subsidiaries. Therefore, we have grouped the sample by firm size and country development level, considering the characteristics of different companies and different countries. The results are shown in Table 5. The first two columns are the results of grouping by firm size, where column 1 shows the results of large-scale firms while column 2 represents small-scale firms. The results show that the coefficients on diversification of overseas subsidiaries remain significantly positive, implying that our regression results are reliable and that the effect of diversification of overseas subsidiaries on the innovation performance of the parent company is not disturbed by the size of the firms themselves. This concern of reverse causation can be eliminated. What is more, the last two columns show the results of grouping by the country development level, where column 3 displays the results of developed countries and regions while column 4 represents the developing areas. The results show that the coefficients of the explanatory variable are not significant but still active. Therefore, this finding remains important, with the coefficients remaining consistent for firms from countries and regions at different development levels, and this consistency corroborates to some extent the plausibility that the diversification of overseas subsidiaries has a positive effect on the innovation performance of the parent company. Additionally, in order to mitigate the concern over the potential reverse causality of parent firm innovation performance affecting the diversification of overseas subsidiaries, we have conducted a differences-in-differences treatment. Treat represents the treatment group, with a value of 1 assigned to firms that implemented decisions to diversify their overseas subsidiaries, indicating they were subjected to the policy shock. Conversely, firms without overseas subsidiary diversification were designated as the control group, with a value of 0. Post denotes the treatment period, where firms in the treatment group experienced the policy shock only during this period, assigned a value of 1 if the firm entered the treatment period and 0 otherwise. The coefficient of the interaction term between Treat and Post is of particular interest to us. The results in column 5 of Table 5 show that the coefficient of this interaction term is statistically significant at the 5% level, suggesting diversification of overseas subsidiaries significantly improves the innovation performance of the parent company, further validating the reliability of our findings.

**Table 5:** Results of endogenous treatment.

	(1)	(2)	(3)	(4)	(5)
Diverse	0.516^*^	0.528^*^	0.383	0.119	
	(0.277)	(0.295)	(0.390)	(0.189)	
Treat * Post					0.474^**^
					(0.189)
Age	0.646	0.545	2.009^**^	−0.498	−0.022
	(0.656)	(0.501)	(0.900)	(0.391)	(0.333)
Size	0.237	−0.029	−0.080	0.193	−0.004
	(0.213)	(0.158)	(0.181)	(0.172)	(0.107)
Productivity	0.250	−0.008	−0.025	0.302	0.031
	(0.207)	(0.119)	(0.195)	(0.202)	(0.099)
RD_intensity	0.013	−0.181	−0.125	0.011	−0.006
	(0.162)	(0.279)	(0.178)	(0.136)	(0.099)
Manage	0.120	−0.029	−0.214	0.085	0.011
	(0.222)	(0.055)	(0.212)	(0.158)	(0.046)
State_owned	0.316	0.029	0.137	0.098	0.184
	(0.418)	(0.286)	(0.659)	(0.381)	(0.313)
Experience	0.062	−0.082^*^	0.070	0.018	−0.029
	(0.044)	(0.045)	(0.057)	(0.044)	(0.115)
Internationalization	0.692	−0.375	−0.268	0.489	0.007
	(0.553)	(0.561)	(0.570)	(0.440)	(0.235)
Distance	0.001	0.003	−0.024	−0.009	−0.015
	(0.004)	(0.006)	(0.015)	(0.011)	(0.100)
Oversea_lay	0.120	0.061	0.247^***^	−0.036	0.211^*^
	(0.075)	(0.098)	(0.095)	(0.089)	(0.108)
Domes_lay	−1.855^*^	0.194	1.373^***^	−0.632^*^	1.350^**^
	(1.097)	(0.373)	(0.518)	(0.374)	(0.655)
Year	YES	YES	YES	YES	YES
Province	YES	YES	YES	YES	YES
Industry	YES	YES	YES	YES	YES
Firm	YES	YES	YES	YES	YES
Constant	21.904	−2.535	2.346	0.699	6.287
	(13.286)	(2.214)	(2.535)	(1.916)	(4.309)
R-squared	0.765	0.769	0.724	0.809	0.661
*N*	2,385	470	1,172	1,683	1,534

*, ** and *** denote 10, 5 and 1% significance levels respectively, with standard errors in parentheses.

### 5.4 Mechanism test

The diversification of overseas subsidiaries may impact the innovation performance of the parent company through the spillover effects of innovation capabilities channel. We aim to substantiate this mechanism by demonstrating the impact of diversification of overseas subsidiaries on the spillover of innovation capability and elucidating the significance of innovation spillover on firm innovation performance. There is already an abundance of literature demonstrating the crucial role of innovation spillovers in enhancing firms’ innovation performance (Kafouros et al., 2012; Ramadani et al., 2017; Audretsch and Belitski, 2020; Sun et al., 2021; Battistella et al., 2023; Wang et al., 2023; Anwar et al., 2024; You et al., 2024). When the innovation capabilities spill over, the firm can more effectively utilize the acquired knowledge and technology, applying them to improve products, services, or business processes. This spillover effect enables firms to introduce more competitive products or services to the market, thereby increasing market share and profitability. Additionally, the spillover of innovation capabilities can also stimulate innovation activities in other firms, fostering the formation of an innovation ecosystem that further drives the development of entire industries or regions. Therefore, the spillover of innovation capabilities not only directly enhances firms’ own innovation performance but also contributes to promoting higher-level innovation spillovers, further fueling the growth of firms’ innovation performance. Following Jiang (2022), our main interest is to ascertain whether there exists the causality between the diversification of overseas subsidiaries and the spillover of innovation capabilities. Therefore, we construct the regression model as shown in Formula (3):


(3) $$\begin{array}{l} \text{Spillove}r_{it}=\beta_{0}+\beta_{1}\text{Divers}e_{it}+\beta_{2}\text{Oversea}\_{\text{lay}}_{it}\\ +\beta_{3}\text{Domes}\_{\text{lay}}_{it}+X_{it}+\left\{F\right\}+\varepsilon_{it}\; \end{array}$$

where $\text{Spillove}r_{it}$ represents the spillover effect of innovation capabilities. $\beta_{1}$ is the primary interest to us. If it is significantly positive, it indicates that the diversification of overseas subsidiaries has a positive effect on the spillover of innovation capabilities. Taking into account data availability, we chose four indicators to represent the spillover of innovation capabilities: per capita profit rate, the proportion of high-tech products, the number of patent applications by residents, and the number of patent applications by non-residents. The corresponding results are presented in columns 1 to 4 of Table 6, respectively. It is obvious that the coefficients of the explanatory variable are all significantly positive, indicating that the causal relationship holds true. Multinational corporations expand their market and resource acquisition channels through diversifying their overseas subsidiaries, thereby increasing profitability and consequently enhancing the per capita profit rate. This augmented profit can be allocated toward greater investment in research and development, as well as the acquisition of high-tech products and technologies, thereby facilitating the formation and accumulation of innovative capabilities. Moreover, favorable intellectual property protection environment and patent policies enable companies to better safeguard their innovative achievements, strengthening their own innovation capabilities and driving the spillover of innovative capabilities. In conclusion, it can be inferred that the spillover of innovation capabilities indeed serves as the mechanism through which diversification of overseas subsidiaries drives the enhancement of parent company’s innovation performance.

**Table 6:** Results of mechanism test.

	(1)	(2)	(3)	(4)
Diverse	79.304^**^	45.446^*^	816.050^***^	758.368^***^
	(35.953)	(26.446)	(106.662)	(59.250)
Age	−0.120	−0.347	−1.125	−0.072
	(0.311)	(0.441)	(1.205)	(0.623)
Size	−0.262^**^	0.071	−0.229	−0.005
	(0.101)	(0.187)	(0.219)	(0.210)
Productivity	0.832^***^	0.064	0.142	0.267
	(0.128)	(0.125)	(0.280)	(0.199)
RD_intensity	0.298^**^	−0.024	−0.396	−0.151
	(0.126)	(0.102)	(0.430)	(0.192)
Manage	−0.021	−0.046	−0.293	−0.135
	(0.042)	(0.047)	(0.270)	(0.133)
State_owned	0.365	−0.054	−0.344	0.199
	(0.305)	(0.194)	(0.647)	(0.516)
Experience	−0.037	0.206	3.922^***^	3.553^***^
	(0.050)	(0.201)	(0.665)	(0.331)
Internationalization	0.572^***^	0.050	−0.409	−0.062
	(0.198)	(0.314)	(0.546)	(0.309)
Distance	0.077	−0.145^*^	0.095	0.092
	(0.078)	(0.085)	(0.258)	(0.095)
Oversea_lay	−0.063	0.056	−0.159	−0.156
	(0.070)	(0.086)	(0.210)	(0.213)
Domes_lay	1.391^***^	−0.136	−6.897^***^	−8.372^***^
	(0.492)	(0.188)	(0.659)	(0.305)
Year	YES	YES	YES	YES
Province	YES	YES	YES	YES
Industry	YES	YES	YES	YES
Firm	YES	YES	YES	YES
Constant	−32.435^**^	1.413	−54.637^***^	−30.481^***^
	(14.479)	(2.657)	(17.387)	(8.941)
R-squared	0.839	0.858	0.963	0.964
*N*	1,378	258	247	246

*, ** and *** denote 10, 5 and 1% significance levels respectively, with standard errors in parentheses.

### 5.5 Moderating effect

In this part, we examine the moderating effects of overseas investment layout and domestic investment layout. The results are shown in Table 7. Column 1 only adds the interaction term between diversification of subsidiaries and overseas investment layout based on the baseline regression. Under this model, the coefficient of the explanatory variable remains significantly positive, as well as the coefficient of the interaction term. This suggests that the overseas investment layout of multinational corporations actually plays a positive moderating role on the main effect. This finding shows that our previously proposed hypothesis 2a is not valid. Besides, column 2 only adds the interaction term between diversification of subsidiaries and domestic investment layout based on the baseline regression. And column 3 adds the two interaction terms mentioned in the two previous columns. Comparing the first three columns we can find that the coefficients of the interaction terms of overseas subsidiary diversification and overseas investment layout maintain significantly positive, which further confirms that overseas investment layout can deepen the facilitating effect of subsidiary diversification on the innovation performance of the parent company. Moreover, considering the heterogeneity of host countries’ level of development and the characteristics of firms themselves, we have also divided the sample according to whether or not the destinations are developed countries and whether or not the firms are state-owned enterprises. The corresponding regression results are shown in the remaining four columns. Column 4 shows the results in developed countries and regions, while column 5 shows the results in developing areas. Column 6 represents state-owned enterprises and column 7 represents non-state-owned enterprises. Comparing the data in the column 4 and column 5, we can find that the coefficient of the explanatory variable in column 4 is significantly negative, while the coefficient of the interaction term of the diversification and domestic investment layout is significantly positive; the coefficient of the explanatory variable in column 5 is also negative, while the coefficient of the interaction term of the diversification and overseas investment layout is significantly positive. These findings suggest that domestic investment layout plays a positive moderating role or even plays a substitution effect in samples where the investment destination is a developed area, while overseas investment layout plays a positive moderating role in samples where the investment destination is a less developed area. Furthermore, by comparing the results in column 6 and column 7, we have found that in the sample of state-owned enterprises, the coefficients of both interaction terms are significantly positive, but the coefficient of the domestic investment layout is larger, suggesting that it plays a more pronounced and positive moderating role; whereas in the sample of non-state-owned enterprises, the overseas investment layout plays a more important substitutive moderating role.

**Table 7:** Results of moderating effect.

	(1)	(2)	(3)	(4)	(5)	(6)	(7)
Diverse	24.509^***^	−21.501	−13.667	−91.330^***^	−249.677	−72.555^*^	−10.835
	(5.749)	(43.514)	(43.089)	(30.941)	(195.333)	(37.442)	(47.000)
Age	0.511	0.443	0.511	2.145^**^	−0.449	0.344	0.461
	(0.555)	(0.560)	(0.555)	(0.874)	(0.386)	(0.908)	(0.596)
Size	0.053	0.082	0.053	−0.121	0.170	0.427	−0.023
	(0.137)	(0.139)	(0.137)	(0.178)	(0.167)	(0.281)	(0.164)
Productivity	0.192	0.217	0.192	−0.054	0.274	0.744^**^	0.093
	(0.150)	(0.154)	(0.150)	(0.187)	(0.199)	(0.296)	(0.144)
RD_intensity	−0.051	−0.049	−0.051	−0.115	0.002	0.493	−0.124
	(0.112)	(0.116)	(0.112)	(0.168)	(0.133)	(0.349)	(0.129)
Manage	0.032	0.037	0.032	−0.191	0.074	−1.539^***^	0.069
	(0.137)	(0.144)	(0.137)	(0.203)	(0.148)	(0.565)	(0.115)
State_owned	0.333	0.335	0.333	0.202	0.071	0.143	23.728
	(0.357)	(0.351)	(0.357)	(0.658)	(0.381)	(0.784)	(17.767)
Experience	0.042	0.050	0.042	0.069	0.011	0.053	0.037
	(0.039)	(0.042)	(0.039)	(0.054)	(0.042)	(0.080)	(0.039)
Internationalization	0.302	0.335	0.302	−0.295	0.423	−0.844	0.368
	(0.370)	(0.397)	(0.370)	(0.563)	(0.395)	(1.110)	(0.382)
Distance	0.008^*^	0.002	0.008^*^	−0.013	−0.011	0.004	0.006
	(0.005)	(0.002)	(0.005)	(0.017)	(0.010)	(0.006)	(0.007)
Oversea_lay	−0.189	0.102	−0.189	−0.030	−0.324^**^	−0.222	−0.208
	(0.130)	(0.072)	(0.130)	(0.178)	(0.150)	(0.221)	(0.139)
Domes_lay	0.430	−0.953	−0.763	0.775^**^	−4.823	−0.092	−0.476
	(0.985)	(0.710)	(0.702)	(0.310)	(3.675)	(0.142)	(0.645)
Diverse_oversea	2.206^***^		2.206^***^	1.678	2.357^**^	1.383^*^	2.699^***^
	(0.814)		(0.814)	(1.129)	(0.972)	(0.828)	(0.773)
Diverse_domestic		3.313	2.386	7.242^***^	136.173	7.371^**^	1.966
		(2.716)	(2.720)	(2.104)	(109.124)	(3.239)	(2.938)
Year	YES	YES	YES	YES	YES	YES	YES
Province	YES	YES	YES	YES	YES	YES	YES
Industry	YES	YES	YES	YES	YES	YES	YES
Firm	YES	YES	YES	YES	YES	YES	YES
Constant	−2.668	−5.893	−4.942	15.572^**^	5.181	−31.207^*^	−1.771
	(6.158)	(4.923)	(4.804)	(6.183)	(3.828)	(15.846)	(2.705)
R-squared	0.760	0.757	0.760	0.727	0.812	0.798	0.765
*N*	2,855	2,855	2,855	1,172	1,683	878	1977

*, ** and *** denote 10, 5 and 1% significance levels respectively, with standard errors in parentheses.

## 6 Conclusion

Based on data from Chinese industrial enterprises database and the investment list from 2000 to 2013, this study examines the impact of diversification of overseas subsidiaries on the innovation performance of parent company. In contrast with existing research, this study takes the diverse, rather than single, subsidiary functions as the main research target. In addition, our research also explores the moderating effects of overseas investment layout and domestic investment layout. Our main findings will be illustrated as follows.

Firstly, our empirical results indicate that diversification of multinationals’ overseas subsidiaries does have a significant positive impact on the innovation performance of the parent company through the spillover effect of innovation capabilities. This conclusion is supported by robustness tests and endogenous treatments, which allow us to have a high level of confidence in the reliability of the results. The discovery not only provides strong support for existing theories at the empirical level, but also provides practical guidance for corporate strategic decision-making. From a theoretical perspective, our findings further enrich the understanding of the relationship between strategic diversification and innovation performance of multinationals. The conventional view holds that multinationals invest abroad only to gain markets and resources. However, our study reveals the limitations of this view, especially in contexts involving diversification of overseas subsidiaries. Compared to single acquisition of markets and resources, diversification of overseas subsidiaries focuses more on the diversity of multinationals’ investments across regions and industries and the functions undertaken by different subsidiaries. The diversification strategy aims to reduce the firm’s risk exposure and exploit opportunities across different regions and industries, thereby improving the overall competitiveness of the parent company. While diversification strategies may pose risks such as capital pressure, management challenges and market risks, our findings indicate that despite increasing the investment size and managerial complexity of multinationals, diversification of overseas subsidiaries positively impacts firms’ innovation performance. We find that diversification strategies help multinationals to enhance their innovation capabilities and strengthen their competitive advantages. Therefore, our study encourages multinationals to continue to focus on investment diversification and adopt appropriate risk management measures when formulating diversification strategies to better achieve innovation performance enhancement.

Secondly, our study finds that overseas investment layout and domestic investment layout play an important role in moderating the relationship between diversification of overseas subsidiaries and the innovation performance of the parent company. Specifically, both overseas and domestic investment layout can deepen the contribution of overseas subsidiary diversification to the innovation performance of the parent company, but certainly there are also differences between them. On the one hand, the domestic investment layout shows a positive moderating effect and even a substitution effect when the investment destination is a developed area. Because in developed regions, domestic investment layout may provide multinationals with a more stable and reliable innovation environment, and therefore, even if the risks resulting from diversification of overseas subsidiaries are high, domestic investment layout can compensate for the negative effects by optimizing the allocation of resources and reducing the level of risk. On the other hand, in the case where the investment destination is a less developed area, the overseas investment layout shows a positive moderating effect. This can be due to the fact that in less developed areas, overseas investment layout provides multinationals with more innovation opportunities and helps multinationals better utilize local markets and human resources, which further enhances the facilitating effect of overseas subsidiary diversification on innovation performance. In addition, our study identifies the differences in the moderating role of domestic and overseas investment layout between state-owned enterprises and non-state-owned enterprises. In the sample of state-owned enterprises, we observe that both domestic and overseas investment layout play a positive moderating role, but the role of domestic investment layout is more crucial. This may be due to the fact that state-owned enterprises have stronger resource advantages and policy support in the domestic market, enabling them to more effectively utilize domestic investment layout to promote innovation performance. On the contrary, as for non-state-owned enterprises, overseas investment layout shows a more important substitutive moderating role. This may be due to the fact that non-state-owned enterprises rely more on overseas markets to access innovation resources and opportunities and less on domestic investment layout. These findings can provide an important reference to further understand the innovation strategy choices of different types of firms, as well as a more accurate guidance for multinationals in making strategic decisions.

The research also has limitations. Our study primarily faces limitations in data availability and coverage. While the baseline regression primarily utilizes the Chinese industrial enterprises database, it may have evident temporal constraints, potentially inadequately capturing the recent changes. Although we have extended the sample period to the most recent years using data from listed companies in the robustness test, the data from listed companies can only represent the investment behaviors of some large enterprises, thus lacking universality and representativeness to some extent, as they are unable to reflect a broader condition. Therefore, future research can explore the integration of additional data sources, such as industry statistics, survey data, or other international databases, to enhance the coverage and representativeness of the study’s data, thereby achieving a more comprehensive understanding of the impact of diversification of overseas subsidiaries on the innovation performance of parent companies.

## Data availability statement

The raw data supporting the conclusions of this article will be made available by the authors, without undue reservation.

## Author contributions

SW: Conceptualization, Data curation, Formal analysis, Methodology, Resources, Software, Validation, Writing – original draft, Writing – review & editing. XC: Methodology, Resources, Validation, Writing – review & editing. JY: Conceptualization, Investigation, Supervision, Writing – review & editing. CZ: Funding acquisition, Project administration, Writing – review & editing.

## Footnotes

^1^ https://www-file.huawei.com/minisite/media/annual_report/annual_report_2022_en.pdf
